# A multi-faceted intervention to implement guideline care and improve quality of care for older people who present to the emergency department with falls

**DOI:** 10.1186/1471-2318-11-6

**Published:** 2011-01-31

**Authors:** Nicholas Waldron, Ian Dey, Yusuf Nagree, Jianguo Xiao, Leon Flicker

**Affiliations:** 1 Department of Rehabilitation and Aged Care, Sir Charles Gairdner Hospital, Perth, Australia; 2 Department of Rehabilitation and Aged Care, Armadale Kelmscott Memorial Hospital, Perth, Australia; 3 Department of Emergency Medicine, Armadale Kelmscott Memorial Hospital, Perth, Australia; 4 Fremantle Hospital Emergency Medicine Research Unit, Discipline of Emergency Medicine, University of Western Australia, Perth, Australia; 5 Epidemiology Branch, Department of Health Western Australia, Perth, Australia; 6 Western Australian Centre for Health and Ageing, School of Medicine and Pharmacology, University of Western Australia, Perth, Australia

## Abstract

**Background:**

Guidelines recommend that older people should receive multi-factorial interventions following an injurious fall however there is limited evidence that this is routine practice. We aimed to improve the delivery of evidence based care to patients presenting to the Emergency Department (ED) following a fall.

**Methods:**

A prospective before and after study was undertaken in the ED of a medium-sized hospital in Perth, Western Australia. Participants comprised 313 community-dwelling patients, aged 65 years and older, presenting to ED as a result of a fall. A multi-faceted strategy to change practice was implemented and included a referral pathway, audit and feedback and additional falls specialist staff. Key measures to show improvements comprised the proportion of patients reviewed by allied health, proportion of patients referred for guideline care, quality of care index, all determined by record extraction.

**Results:**

Allied health staff increased the proportion of patients being reviewed from 62.7% in the before period to 89% after the intervention (P < 0.001). Before the intervention a referral for comprehensive guideline care occurred for only 6/177 (3.4%) of patients, afterwards for 28/136 (20.6%) (difference = 17.2%, 95% CI 11-23%). Average quality of care index (max score 100) increased from 18.6 (95% CI: 16.7-20.4) to 32.6 (28.6-36.6).

**Conclusions:**

A multi-faceted change strategy was associated with an improvement in allied health in ED prioritizing the review of ED fallers as well as subsequent referral for comprehensive geriatric care. The processes of multi-disciplinary care also improved, indicating improved care received by the patient.

## Background

Widespread deficits in the delivery of quality health care to older people have been highlighted [1]. These gaps are greater for older people with geriatric syndromes (e.g. falls, incontinence, cognitive impairment) compared with older people with general medical conditions (e.g. hypertension, diabetes) and fall well short of acceptable levels [2]. This is of concern as older patients, who often have limited physiological reserve and high health care utilization, may gain the greatest health benefits when optimal care is delivered. For falls prevention the evidence for these interventions has been highlighted in multiple guidelines [3,4].

Over one third of community-dwelling older people fall each year and many who have sustained the most serious falls attend the Emergency Department (ED) with falls accounting for 18% of presentations to ED in older adults [5,6]. The personal cost of injurious falls is well established with patients developing a fear of falling, increased frailty, reduced quality of life and loss of independence [7-9]. In addition, the already significant economic costs are set to rise dramatically [10,11].

The recommended care for community-dwelling patients following an injurious fall is multi-factorial intervention (e.g. exercise program, home modification and medication changes) based on an individual assessment [3,4]. There is evidence that appropriate care is not delivered when assessed using retrospective patient interviews [12]. When prospectively followed only 3.7% of people presenting with a fall to a Canadian ED received guideline care, and subsequently patients demonstrated worsening falls risk profiles after 6 months [13].

Traditionally, the primary focus of ED in attending patients following falls is the assessment and acute management of injuries, however optimal care should include appropriate referral for prevention of further falls [14]. Barriers to referral cited by providers included patient non-compliance, lack of physician availability, reimbursement limitations and lack of availability of relevant services in the community [15]. We aimed to identify strategies to improve referrals for comprehensive geriatric care, and to determine if these strategies were effective in improving the process of care delivery.

## Methods

### Background and Setting

The setting was Armadale Kelmscott Memorial Hospital ED based in a 280 bed general hospital in Western Australia (WA). The ED has approximately 44,000 patient visits per year. The Geriatric Medicine Department provides outpatient geriatrician consultation on request, outpatient appointments for allied health services and an occupational therapy home visiting service. A previous audit in 2005/6 found a maximum of 4.9% of patients presenting to ED with a fall may have been referred for allied health review with no referrals for geriatrician review. The other service providing comprehensive geriatric care was "rehabilitation in the home" (RITH), a service targeting patients normally requiring admission who could safely be managed at home with multi-disciplinary care and availability of geriatrician review.

In October 2007, as part of a state-funded hospital diversion program to avoid unnecessary hospital admissions, care co-ordination teams (CCT) based in ED's were introduced. CCT were multi-disciplinary, most commonly including physiotherapists, occupational therapists (OT) and social workers, working standard hours and mornings during weekends. Their primary role was to screen older people attending ED to identify vulnerable older patients for more comprehensive assessment and referral, prioritizing those who could be discharged. This service development was unrelated to the research project and occurred after project conception.

### Strategy for change

The study was under-pinned by concepts for changing clinical practice and knowledge translation [16,17]. Target groups included clinical staff involved in referring or receiving referrals for ED fallers and key executive staff. Local barrier analysis was undertaken using informant interviews and focus groups with change strategies linked to the identified barriers [18,19]. Performance indicators to measure change were developed and included the proportion of discharged ED fallers reviewed by CCT and the proportion of discharged ED fallers referred for guideline care.

#### Engaging the target group

Formation of a senior level multi-disciplinary steering group occurred at project inception to assist project design and support change strategies including obtaining funding for service development. The patient journey was mapped following semi-structured interviews with key ED, nursing, allied health and quality improvement staff. The resulting process map and baseline performance indicators were presented to two focus groups (30 May 2008), along with the key guideline recommendation: "following treatment for an injurious fall, older people should be offered a multi-disciplinary assessment to identify and address future risk and individualized intervention, aimed at promoting independence and improving physical and psychological function" [4]. The groups were then asked to identify enablers and barriers to closing the evidence-practice gap (documented on a white board) with discussion undertaken as to how to improve care. The first was attended by 9 practitioners (ED nurses - 8, quality improvement officer - 1) the second by 8 practitioners (emergency physicians - 2, geriatrician - 1, occupational therapists - 2, physiotherapists - 2, social worker - 1). Key barriers were identified and categorized through review of interview and focus group documentation (written notes). Patient related barriers were considered out of scope for the project.

#### Barrier Analysis

The identified barriers existed at three levels (see Table 1).

**Table 1:** Barrier Analysis Determined by Interviews and Focus Groups with a Multi-faceted Intervention to Overcome these Barriers

Barriers	Intervention	Description
*Individual:*		
Access to and working knowledge of guideline recommendation	Referral pathway with resources and education	- A referral pathway adapted to local services was developed - A single education session was delivered to CCT with a pathway, neck pendant and resource folder including prefilled referrals
Overly optimistic about performance	Audit and feedback to CCT	- Audit and feedback was provided on two occasions to CCT staff with key performance indicators presented and discussion about how to improve care

Group:		
Lack of time Overlapping roles in ED Unclear after hours processes	CCT role clarified during review of processes for ED fallers	- Processes revised to avoid blocks in patient flow - Agreement that CCT would prioritize and have primary responsibility to refer ED fallers - CCT physiotherapy and OT to take lead referral role - Process for daily screening of patients presenting after hours with subsequent follow-up telephone calls
Lack of peer support	New "OT falls specialist" role	- OT falls specialist role provided feedback and mentoring

*Organizational:*		
Increased workload for existing services No community-based exercise programme	Expansion of falls services	- Geriatric Medicine Department employed an OT falls specialist and increased geriatrician time - Community-based group exercise program commenced

CCT = care co-ordination teams, OT = occupational therapy, ED = emergency department.

1) Individual clinicians - These themes included lack of knowledge of the guideline recommendation, perception that ED fallers may not require guideline level care and that low risk patients may not require referral at all and that appropriate identification and referrals already occurred.

2) Group - These themes included the belief held by ED staff that falls prevention was not "core business" with many competing interests for staff time including pressure to reduce ED length of stay. There was concern regarding overlapping roles between ED nursing and CCT staff in managing falls patients without a clear care pathway, especially after hours. CCT were concerned that they were only resourced to review 30% of all older adults presenting to ED. A lack of clinical peer support and clear role delineation was noted within the CCT team.

3) Organizational - There were concerns that increased referrals may overtax existing geriatric medicine services and cause delays in other parts of the hospital system. The managers noted a lack of local community-based falls prevention exercise programs. There was concern that RITH was not providing care consistent with guidelines.

#### Linking change strategies to barriers

The change strategies were multi-faceted designed to deal with the identified barriers (see Table 1).

**Figure 1:**
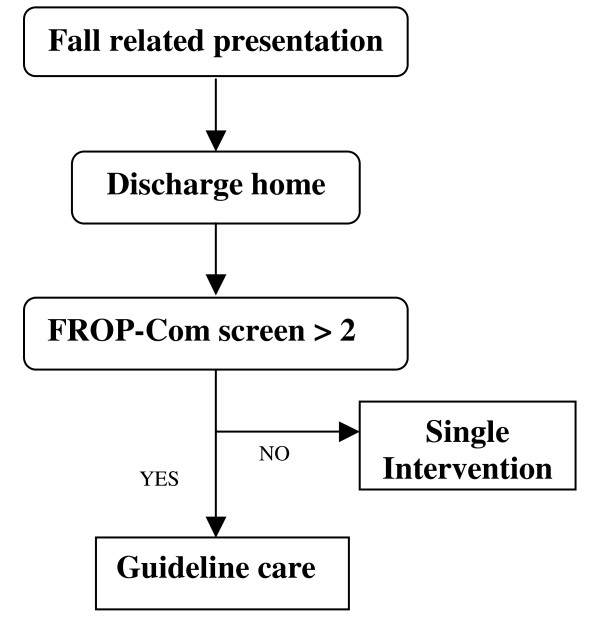
**Stratified Referral Pathway for Community Dwelling Older Patients Presenting to ED Following a Fall**. Clinician and patient preferences should be taken into account when deciding referral option. FROP-Com screen includes 3 items (falls history, function and balance) with a maximum score of 9.

1) Interventions directed towards individual health practitioners - A referral pathway was developed, with reference to the steering group (see Figure 1). The pathway stratified patients using the "falls risk for older people in the community (FROP-Com) screening tool" with a score greater than two used to identify a high risk group more likely to benefit from comprehensive assessment [20-22]. The pathway was a guide to support evidence based decision making, intended to be influenced by clinical judgement and patient preference [23]. The pathway design was simple to minimize additional ED staff input and the potential impact on ED length of stay. The pathway was introduced during a single case based education session for CCT along with the provision of resources (neck pendant and resource file). Audit and feedback of performance indicators were provided at 1 and 3 months from implementation during meetings with CCT. At these sessions, ways that CCT could improve care were explored.

2) Group orientated interventions - The roles within the CCT were clarified, with physiotherapy and/or occupational therapy taking the lead roles. A process for after hours ED fallers was agreed. An "OT falls specialist" role (based in the Geriatric Medicine Department) was introduced to support timely clinical follow-up, monitor the referral process and provide informal feedback and mentoring to CCT.

3) Organizational interventions - The geriatric medicine department was expanded with additional geriatrician time (1 day per week) and occupational therapy time (2 days per week). Referred patients were managed to reflect the care provided in the PROFET trial with OT home visit followed by geriatrician review and subsequent referral as indicated [24]. RITH was strengthened with physiotherapists trained to deliver individualised Otago exercise programs [25]. A community-based physiotherapy lead group exercise program was set up based on successful exercise interventions [25,26].

### Patient selection and data collection

The target population comprised older people aged greater than 65 years of age (or Aboriginal and Torres Strait Islanders age over 45 years of age) presenting to ED as the result of a fall. A patient list was produced from the Emergency Department Information System (EDIS version 9.36, iSOFT Plc), with patients excluded if they were admitted, transferred or resided in a residential care facility. The remaining subjects' triage entry and diagnosis was read by the primary author (NW) and excluded if the description did not meet the standard definition of a fall or falls prevention was not clinically indicated (i.e. clear medical cause or palliative condition) [27]. Clear medical cause included seizure, stroke or an alcohol-related fall. Patients with definite syncope and delayed presentations >4 days were excluded, the later to minimize the number with multiple presentations. The before-intervention period was 1 December 2007 to 30 June 2008, the post-intervention period was 1 September 2008 to 31 March 2009 with 2 months for the intervention to be established.

Data sources included EDIS and the hospital WA Hospital Morbidity Data System which provided demographic, hospital utilization and co-morbidity data (Charlson co-morbidity index) [28]. In addition case notes were audited to determine performance in terms of referral patterns and quality of care. Audit items were extracted up to 4 months after first presentation to ED.

### Evaluating outcomes

Two indicators were used to measure change. The first was the proportion of the target patient population reviewed by CCT either in person or by follow-up telephone call. The second was the proportion of the target patient population referred for guideline care. Guideline care was defined as multi-factorial interventions based on individualized assessment. Local referral options meeting guideline care criteria included the Geriatric Medicine Department (providing occupational therapy home visit followed by geriatrician outpatient review), referral to RITH or referral by CCT to two single interventions (multiple referrals). A third indicator was used to capture the proportion referred for a single intervention. A single intervention was defined as an intervention which addressed one category of risk factor. This included referral for an exercise program (community or hospital based) or occupational therapy home visit.

Quality of care was determined based on a score developed by the Clinical Effectiveness and Evaluation Unit (CEEU) of the Royal College of Physicians, as a component of a large audit used to review care delivered to older people presenting to ED with fragility fractures [29]. The composite score is included under the "Multi-factorial risk assessment and intervention" domain and has 15 items, weighted to give a maximum score out of 100. Items include the falls history and examination (vision, cognition, postural blood pressure and ECG) and interventions (exercise and occupational home visits).

### Statistical analysis

Multiple databases were merged using the unique patient identifier allocated within the Open Patient Administration System in Western Australia. The data were analysed using PASW Statistics version 17.0. Comparisons of patient characteristics and processes of care, before and after interventions, were conducted using independent *t*-tests, Chi square tests and Mann-Whitney U tests depending on the nature and distribution of data. An autoregressive integrated moving average (ARIMA) model was used to examine the trend of monthly ED presentations of patients from December 2007 to March 2009. The model was used to assess whether the intervention was effective in increasing guideline care taking into account CCT staffing levels as measured by full time equivalent (FTE) staff. For this model the intervention date was set at the beginning of the intervention period as this is when the majority of the interventions occurred. Approval for the project was granted by the Human Research Ethics Committee of South Metropolitan Area Health Service, Western Australia.

## Results

**Figure 2:**
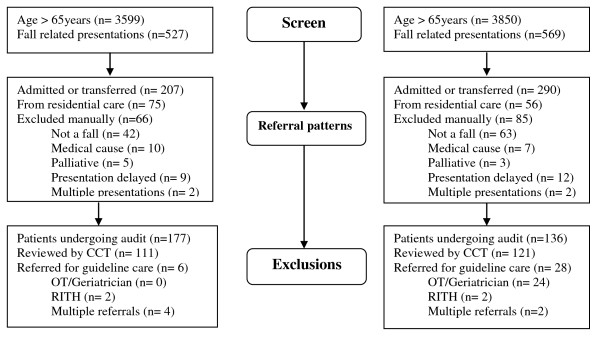
Flow Chart for Patient Selection and Referrals for Guideline Care  (RITH = rehabilitation in the home, CCT = care co-ordination teams, OT = occupational therapy).

During the study period 14.7% of presentations in older people were fall related. Of the 1,096 presenting with a fall, 54.7% were discharged directly from ED. After exclusions, 313 patients met enrolment criteria being both community-dwelling and presenting with a clinical scenario where secondary falls prevention was indicated (see Figure 2). The baseline characteristics of the before and after groups showed no difference in terms of age, sex, time of presentation, locality, indigenous status, time spent in ED, co-morbidities or the level of pre-existing care (see Table 2).

**Table 2:** Basic Characteristics of the Before and After Intervention Cohorts

Measure	Before (n = 177)	After (n = 136)	P value
Age in years, Mean ^36^	75.86 (7.84)	76.54 (8.07)	0.455
Sex, N (%)			
Male	54 (30.5)	40 (29.4)	0.834
Female	123 (69.5)	96 (70.6)	
Duration in ED in hours, Mean ^36^	3.27 (1.61)	3.73 (2.73)	0.063
Attended during CCT hours, Yes (%)	99 (58.9)	85 (62.5)	0.242
From local catchment area, Yes (%)	142 (80.2)	117 (86.0)	0.178
Presentation period, N (%)			
Daytime (0800 - 1700)	113 (63.8)	94 (69.1)	0.536
Evening (1700 - 2400)	53 (29.9)	33 (24.3)	
Overnight (0000 - 0800)	11 (6.2)	9 (6.6)	
Aboriginal patient, Yes (%)	6 (3.4)	5 (3.7)	0.891
Patient with existing care, Yes (%)	13 (7.3)	10 (7.4)	0.998
Charlson co-morbidity index, N (%)			
No co-morbidity	139 (78.5)	109 (80.1)	0.912
1-2 co-morbidities	30 (16.9)	22 (16.2)	
3+ co-morbidities	8 (4.5)	5 (3.7)	
Emergency admissions in past 3 years, Yes (%)	40 (22.6)	25 (18.4)	0.362

SD = standard deviation, ED = emergency department, CCT = care coordination team.

Table 3 shows that CCT performance improved with the proportion of ED fallers reviewed increasing from 62.7% before to 89% after the intervention (χ^2 ^= 27.646, p < 0.001). The proportion of patients being referred for guideline care also significantly increased from 3.4% (6/177) before to 20.6% (28/136) after the intervention (absolute difference = 17.2%, 95% CI 11-23%, p < 0.001). This improvement was accounted for by an increased rate of referral for combined OT home visit/geriatrician review increasing from 0/177 cases before to 24/136 cases after the intervention (see Figure 2). Although there was a trend towards improved referrals for single interventions, this only reached borderline statistical significance changing from 13% before to 21.3% after the intervention (χ^2 ^= 3.851, p = 0.050). The average quality of care index improved 75.3% from 18.6 (95% CI: 16.7-20.4) to 32.6 (28.6-36.6) out a maximum score of 100. When benchmarked to the CEEU national audit scores, this improvement represented a move from the first quartile to the third quartile of all sites evaluated [29].

**Table 3:** Performance Indicators and Quality of Care Received by ED Fallers being Discharged Home Before and After a Multi-faceted Intervention

Measure	Before (n = 177)	After (n = 136)	P-value
Indicators			
1. Reviewed by CCT, N (%)	111 (62.7)	121 (89)	<0.001
2. Referred for guideline care, N (%)	6 (3.4)	28 (20.6)	<0.001
3. Referred for single intervention, N (%)	23 (13.0)	29 (21.3)	0.050
			
Quality of care score, mean	18.6 (12.5)	32.6 (23.4)	<0.001

SD = standard deviation, ED = emergency department, CCT = care coordination team.

**Figure 3:**
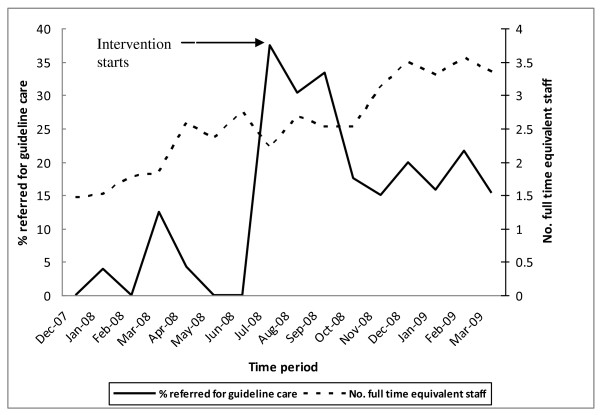
**Percentage of ED Fallers Referred to Guideline Care and Staffing Levels of CCT during the Study Timeframe**.

During the project time-frame, CCT staffing level increased from 2.05 FTE staff members before the intervention to 3.13 FTE staff members after the intervention. The number of FTE staff and the proportion referred for guideline care for each month over the study timeframe are depicted in Figure 3. Time series analysis results showed that the intervention significantly increased the percentage referred for guideline care (t = 2.306, p = 0.044). Modelling via an ARIMA model for the effect of FTE staffing levels indicated that the increase in staff did not seem to have an effect on referrals for guideline care (t = 0.154, p = 0,880).

## Discussion

The multi-faceted strategy was effective in improving the delivery of guideline care to people presenting to ED with falls who were being discharged home. Although the absolute improvement of 17.2% (3.4 - 20.6%) for referral to guideline care may appear small, this is favourable when compared to the median effect of 10% improvement found in a systematic review of guideline dissemination and implementation strategies [30]. The choice of a multi-faceted approach was likely to be important given that identified barriers existed at multiple levels, although such an approach may not always be required for guideline implementation [30]. The low levels of adherence to recommended care at baseline supported the inclusion of audit and feedback where this change strategy is more effective [31]. An often neglected step to optimizing care delivery was the increased falls prevention resources, supporting health care providers to manage the anticipating increased work flow [32]. It is encouraging the multi-disciplinary care index improved, this supports that improved care was actually received by the patient.

The presence of allied health (CCT) in ED addressing the care needs of older patients likely supported a positive outcome. The improved referral rates reflected a change of clinical practice by CCT, both with improved identification of ED fallers and subsequent referral. It is uncertain which component of the intervention affected these changes. The use of a referral pathway is likely to have been an important knowledge tool for busy clinicians [14,15]. Although the choice to stratify ED fallers may have reduced the proportion referred for guideline care, this was felt important to reflect current clinical practice, allow for patient and clinician choice as well as a more cost-effective approach [20,23,33]. The introduction of the "OT falls specialist" role was likely to have influenced change, firstly by providing staff time for the increased clinical workload but also the day to day contact required for building confidence in referrers, to mentor new staff and improve linkages between the emergency and geriatric medicine departments.

Falls prevention following ED attendance requires a complex intervention and has consequently been a challenging area to change clinical practice. Direct referral to hospital-based geriatricians with timely and proactive review is likely to represent optimal care for higher risk patients [24,34]. The translation of this research into practice is highly important both at a personal and societal level: patients will decline physiologically without optimal care [13], and health system costs will escalate if fall rates do not decline [11]. A multi-factorial change strategy, as demonstrated in this study, is likely to be required to improve health service delivery. In addition, careful attention should also be paid to the quality of geriatric care delivered after referral and not simply providing "usual care" [34]. Having allied health in ED as in this study, may support ED physicians that also recognise an increasing role in prevention of recurrent falls [35].

### Limitations

Limitations of this study include its non-randomized design, utilizing a before and after study design which does not exclude certain bias. There were no measured patient outcomes, this program relying on process measures only. The transferability of these results to other settings is uncertain, differences in ED size, local culture or existing inter-departmental relations are likely to be important. The program applicability is uncertain without the presence of dedicated staff in ED attending the post-ED needs of older patients. The short study period has meant program sustainability has not been addressed.

## Conclusions

A multi-faceted change strategy improved referral patterns for ED fallers to comprehensive geriatric care. The processes of multi-disciplinary care also improved indicating better care received by the patient. Components likely to be required include active engagement of involved staff, a locally-adapted referral pathway and audit and feedback of performance indicators (attained from notes audit). The additional resources to expand geriatric medicine services (OT falls specialist and increased geriatrician time) supported meeting the increased workload and improving links between Geriatric and Emergency Departments. The strategy used was dependent on having allied health staff (CCT) based in ED to attend the care needs of older patients.

## Conflict of interest

The authors declare that they have no competing interests.

## Authors' contributions

NW attained funding, participated in study design, data acquisition, data analysis, data interpretation and manuscript preparation. LF supervised the study and participated in study design, data interpretation and revised the manuscript. JX ensured completeness of data collection, performed the statistical analysis and interpretation of data and revised the manuscript. YN and ID participated in study design, data acquisition and manuscript revision. All authors have read and approved the final manuscript.

## Pre-publication history

The pre-publication history for this paper can be accessed here:

http://www.biomedcentral.com/1471-2318/11/6/prepub
